# Liver Transplantation from Voluntary Organ Donor System in China: A Comparison between DBD and DCD Liver Transplants

**DOI:** 10.1155/2019/5736702

**Published:** 2019-05-02

**Authors:** Wei Chen, Dipesh Kumar Yadav, Xueli Bai, Jianying Lou, Risheng Que, Shunliang Gao, Guogang Li, Tao Ma, Ji Wang, Bingfeng Huang, Tingbo Liang

**Affiliations:** ^1^Department of Hepatobiliary and Pancreatic Surgery, The First Affiliated Hospital, Zhejiang University School of Medicine, Hangzhou 310003, China; ^2^Department of Hepatobiliary and Pancreatic Surgery, The Second Affiliated Hospital, Zhejiang University School of Medicine, Hangzhou 310009, China

## Abstract

**Background:**

In China, the cases of liver transplantation (LT) from donation after citizens' death have rose year by year since the citizen-based voluntary organ donor system was initiated in 2010. The objective of our research was to investigate the early postoperative and late long-term outcomes of LT from donation after brain death (DBD) and donation after circulatory death (DCD) according to the current organ donation system in China.

**Methods:**

Sixty-two consecutive cases of LT from donation after citizens' death performed in our hospital between February 2012 and June 2017 were examined retrospectively for short- and long-term outcomes. These included 35 DCD LT and 27 DBD LT.

**Result:**

Subsequent median follow-up time of 19 months and 1- and 3-year graft survival rates were comparative between the DBD group and the DCD group (81.5% and 66.7% versus 67.1% and 59.7%; *P* = 0.550), as were patient survival rates (85.2% and 68.7% versus 72.2% and 63.9%; *P* = 0.358). The duration of ICU stay of recipients was significantly shorter in the DBD group, in comparison with that of the DCD group (1 versus 3 days, *P* = 0.001). Severe complication incidence (≥grade III) after transplantation was identical among the DBD and DCD groups (48.1% versus 60%, *P* = 0.352). There was no significant difference in postoperative mortality between the DBD and DCD groups (3 of 27 cases versus 5 of 35 cases). Twenty-one grafts (33.8%) were lost and 18 recipients (29.0%) were dead till the time of follow-up. Malignancy recurrence was the most prevalent reason for patient death (38.8%). There was no significant difference in incidence of biliary stenosis between the DBD and DCD groups (5 of 27 cases versus 6 of 35 cases, *P* = 0.846).

**Conclusion:**

Although the sample size was small to some extent, this single-center study first reported that LT from DCD donors showed similar short- and long-term outcomes with DBD donors and justified the widespread implementation of voluntary citizen-based deceased organ donation in China. However, the results should be verified with a multicenter larger study.

## 1. Introduction

The shortage of donors compared with the number of patients in need of a transplant is a serious and persisting problem, both worldwide and in China. Moreover, China has long been criticized for commercial and unethical use of organs from executed prisoners among the international community [1]. Otherwise, with the number of death sentences decreasing year by year, if not establishing a voluntary donation system, severe shortage of donor organs is inevitable [2]. However, China, as a responsible associate of international fraternity, published the regulation on human organ transplantation in 2007, which was the milestone to regulate organ transplantation and establish a legitimate and viable voluntary organ donation architecture [3]. Additionally, to enlarge the donor pool, since March 2010, the pilot program on DCD has been carried out and welcomed by the international transplant family [4, 5]. With the success of implementing the voluntary citizen-based organ donation program, all hospitals have terminated using organs from executed prisoners and the civilian organ donation has been the sole source for an organ transplant in China since January 2015 [6].

Unlike that of the international organ donation criteria, vegetative state and brain death remain unclear for many Chinese citizens. Therefore, on the premise of China's legitimate and social structure, China perceived circulatory death as a legitimate standard and added a new class to the international organ donation criteria: organ donation after brain death pursued by circulatory death (DBCD). On this basis, in China, the organ donations after citizens' death were classified as follows: category I: DBD; category II: DCD; and category III: DBCD (similar to Maastricht-IV) [6, 7]. Therefore, for easy understanding, China organ donation categories II and III can be considered together as DCD because the organ in category III is only retrieved after cardiac arrest.

DCD has become an important source of organs in an endeavor to expand the donor pool [8, 9]. However, DCD liver transplantation (LT) is related with higher rates of graft failure and biliary complication in comparison with DBD LT that are related to warm ischemia, early allograft dysfunction, and prolonged cold ischemia time [10, 11]. In contrast, recent studies show that with careful selection of DCD grafts and recipients the survival rates can reach a level comparable with DBD LT [12–15]. In the study by Blok et al., they found that DCD LT has an increased risk for low graft survival compared to DBD LT, but this study did not find any difference in patient's survival between both groups. Moreover, they concluded that DCD allografts with a first warm ischemia time more than 25 minutes are associated with an increased risk for a decrease in graft survival [16]. Results from controlled DCD, however, are encouraging, although most centers reported that graft and patient survival rates were lower than those of LT with DBD grafts.

In China, the cases of LT donation after citizens' death have been increasing every year [6]. However, the research on the outcomes of LT using DCD donor grafts and DBD donor grafts was rare. Up to the time of writing the manuscript, only several research or case reports summarized the experience of LT from DCD donors [17–19]. We have performed 62 cases of LT from donation after citizens' death in our transplant center from February 2012 to June 2017. The objective of our research was to share our experience on citizens' death donor LT and compare the postoperative and long-term outcomes of LT from DBD donors with those from DCD donors under the current organ donation system in China.

## 2. Patients and Methods

### 2.1. Study Population

This retrospective cohort study included all consecutive 62 cases of LT from donation after citizens' death, which were performed at the Second Affiliated Hospital School of Medicine of Zhejiang University from February 2012 to June 2017. Our hospital was the first batch of pilot program in Zhejiang province to start liver transplantation using voluntary citizen-based organ donation from 2012. The data were collected from the China Liver Transplant Registry System, which collects all data prospectively. In this study, patients were divided into two groups indicated by the sort of donors: DBD group (category I, 27 cases) and DCD group (categories II and III, 35 cases). Every case was reviewed and permitted by the institutional review board of our hospital and was consistent with the Declaration of Helsinki [20].

The primary diseases of recipients included hepatocellular carcinoma (HCC) without vascular invasion or metastasis, hepatitis B virus- (HBV-) related cirrhosis, alcoholic cirrhosis, acute liver failure, and other end-stage liver diseases. Hangzhou criteria were used for the selection of recipients with HCC.

### 2.2. Procurement and Allocation of Liver Grafts

The selection and procurement of donors were performed according to the national guidelines for donation after cardiac death in China [7]. Acceptable criteria for donors included age ≤ 65 years old; no positive HIV infection; and no history of drug abuse, untreated systemic sepsis, or malignancy except central nervous system tumors. In brief, after informed consent for organ donation was obtained from the donor's closest relatives, an independent physician from the intensive care unit (ICU) or neurology department was assigned to withdraw life support. Furthermore, after observation of asystole for 2 to 5 minutes, and final declaration of death, organ procurement started immediately in the operating room. Besides, livers were perfused with 4 to 8 L of histidine-tryptophan-ketoglutarate (HTK) solution via both the abdominal aorta and the superior mesenteric vein and then stored in HTK solution at 4°C. Likewise, the graft procurement from DBD was performed according to the standard technique after brain death was declared. Moreover, livers from DBD donors were also perfused via the aortic and the portal system with HTK solution and were preserved in cold storage until transplantation. Donor warm ischemia time (DWIT) was defined as the time from life support removal to perfusion with cold preservation solution. Nevertheless, DWIT occurred only for DCD grafts.

The livers for donation after citizens' death were allocated by the China Organ Transplant Response System (COTRS) [2], and the organ allocation policy was similar to that of the United Network for Organ Sharing (UNOS) [21].

### 2.3. Liver Transplant and Outcomes

After evaluation of the quality of liver grafts by pre- and postflush appearance and frozen section, LT was performed with a standard piggy-back cavo-caval anastomosis. Additionally, postoperative immunosuppressive regimens are comprised of mycophenolate mofetil (MMF), tacrolimus with or without prednisolone. Characteristics of donors, recipients, and operations were collected. In addition, duration of ICU and hospital stay, postoperative complications, graft loss, and patient death were recorded in the course of the follow-up. Postoperative complications were classified by the Clavien-Dindo classification [22]. Initial poor function (IPF) was defined as alanine aminotransferase (ALT) or aspartate aminotransferase (AST) > 1500 IU/L on two consecutive measurements within 72 h after liver transplantation [23]. All 62 cases were followed up posttransplantation, and the median follow-up course was 19 months.

### 2.4. Statistical Analysis

Continuous variables were presented as a median and interquartile range and categorical variables as percentages (%). Mann-Whitney *U* test or Student's *t*-test was performed to examine continuous variables. Categorical variables were examined with Fisher's exact test or Chi-square test. Kaplan-Meier method was used for analysis of patient and graft survivals and with Log Rank test to compare survivals between the DBD and DCD groups. Statistical analysis was performed with Statistical Package for Social Sciences Software version 19.0 for windows (IBM Corporation, Armonk, NY). A *P* < 0.05 was considered statistically significant.

## 3. Results

### 3.1. Donor and Recipient Characteristics

Between the total groups, the donor and recipient median ages were 42 and 52 years, respectively. There was 50 (80.6%) male among 62 donors and 52 (83.9%) male among 62 recipients. A correlation of donor and recipient variables among the DBD group and the DCD group is shown in Tables 1 and 2. Donors in the DBD group had a longer duration of ICU stay (10 versus 6 days; *P* = 0.01) than those in the DCD group. Other clinical characteristics were not significantly different among both groups.

### 3.2. Operative and Postoperative Outcomes

As described in Table 3, patients in the DCD group had a longer anhepatic phase (76 versus 54 minutes; *P* = 0.007) than those in the DBD group. The duration of ICU stay was significantly shorter in the DBD group than in the DCD group (1 versus 3 days; *P* = 0.001). There was no difference in the blood loss, RBC transfusion, cold ischemia time, operation time, ventilation, and morbidity between the 2 groups. Severe complication incidences (≥grade III) after transplantation were comparable between the DBD and DCD groups (48.1% versus 60%; *P* = 0.352). Common postoperative complications are listed in Table 4. Pleural effusion was the most common complication (38.7%). Four recipients (6.4%) had vascular complications: 3 had HAT (4.8%) and 1 had portal vein thrombosis (1.8%). One case (1.8%) had PNF and 1 case (1.8%) had IPF. Other postoperative complications included pulmonary infection 11 (17.7%), ascites 6 (9.7%), renal dysfunction 6 (9.7%), abdominal hemorrhage 6 (9.7%), acute rejection 6 (9.7%), incision infection 3 (4.8%), and bile leakage 1 (1.8%). Otherwise, there were some rare complications such as 2 cases GVHD (3.6%) and 2 cases intestinal perforation (3.6%).

### 3.3. Outcomes of Graft and Patient Survival

**Figure 1:**
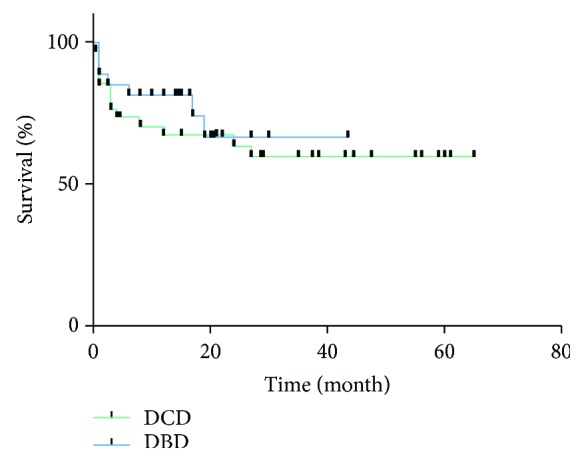
Graft survival in the DCD and DBD groups. *P* = 0.550.

**Figure 2:**
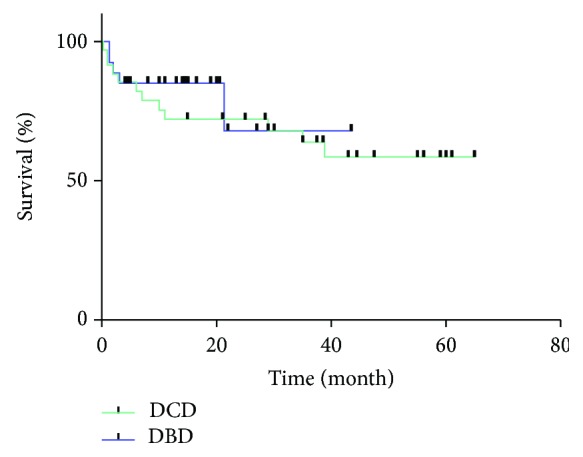
Patient survival in the DCD and DBD groups. *P* = 0.358.

In subsequent median follow-up course of 19 months (range from 0 to 65 months), 21 grafts (33.8%) were lost and 18 recipients (29.0%) were dead. Causes of patient death are shown in Table 5. Malignancy recurrence was the most prevalent reason for patient death (38.8%). Seven cases of recurrence included 6 cases of HCC and 1 case of pNET with liver metastasis. The 1- and 3-year graft survival between DBD and DCD LT was 81.5% and 66.7% vs. 72.2% and 63.9%, respectively. Similarly, 1- and 3-year patient survival rates between DBD and DCD LT were 85.2% and 68.1% vs. 67.1% and 59.7%, respectively. Graft survival and patient survival were not significantly different between the DBD group and the DCD group (*P* = 0.550 and *P* = 0.358) (Figures 1 and 2). During the follow-up period, there were 11 cases of biliary anastomosis stenosis in the whole group (5 cases in the DBD group and 6 cases in the DCD group, *P* = 0.846). Eight cases were placed with stents and 1 case was treated with balloon expansion by endoscopic retrograde cholangiopancreatography. Another 2 cases were treated by percutaneous transhepatic cholangial drainage and conservative treatment, respectively. All 11 cases had good liver function at the time of follow-up.

## 4. Discussion

Since the implementation of the pilot program on voluntary citizen-based deceased organ donation in 2010, China has increased its donor pool year after year [6]. DCD has been playing a vital role in increasing the donor pool; however, the utility of DCD grafts has been a matter of debate and is surrounded by controversies in the transplant community [24, 25]. Additionally, early studies showed lower graft survival for DCD LT compared to DBD LT, considering DCD liver as high-risk grafts [26–28]. Later, several other studies reported identical posttransplant outcomes for DCD and DBD grafts [12, 13]. In our study, the 1- and 3-year graft survival and patient survival rates for the DCD group were identical to the DBD group. It is not completely evident why there is a difference in results between these studies. Probably, it is sensible to accept that patient's characteristics, patient's selection, and surgeon's experience in these studies were not the same and, thus, brought about various results. Furthermore, the reason behind similar overall survival of grafts between DCD and DBD in our study might be that the recipients of DCD grafts were with lower MELD scores, and in contrast, the recipients receiving DBD grafts were with higher MELD scores; however, there was no significant difference in MELD scores between the two groups.

Moreover, as seen in our study, donors in the DBD group had a longer length of ICU stay than those in the DCD group, which may be a result of the nature of the disease in DBD donors. On the other hand, the DCD group had a longer anhepatic phase than the DBD group; apparently, this did not have a negative influence on outcomes. However, other clinical variables of donors and recipients were not significantly different between both groups. Additionally, recipients in the DCD group had a longer anhepatic phase than those in the DBD group; the reason might be by virtue of the more DCD liver transplant was done in the initial series of our study and later on our transplant team got more experience while doing DBD liver transplant; thus, that might have influenced the result.

Nevertheless, the duration of ICU stay was longer in the DCD group, in contrast to that of the DBD group. This result is consistent with previous studies [29, 30]. The probable logic for longer ICU stay in the DCD group might be related to graft warm ischemia time, which may result in the severe cellular breakdown in DCD grafts leading to postreperfusion hyperkalemia, fibrinolysis, hemodynamic instability, and prolonged need for vasopressor and antifibrinolytic support [29, 31–33]. Nonetheless, severe complication incidences (≥grade III) after transplantation were comparable between both groups. Pleural effusion was the most common complication (38.7%), followed by pulmonary infection (17.7%) besides GVHD (3.6%) and intestinal perforation (3.6%) as some of the rare complications among the total number of patients. Likewise, pulmonary complications in our study were similar to other studies reported earlier [34, 35]. Perhaps, considerable causes such as prolonged cold ischemia time, a Piggy-back procedure, hepatic vein outflow obstruction, the presence of portal hypertension without cirrhosis, and prolonged ventilation are some of the main associated variables.

In our analysis, in subsequent median follow-up course of 19 months, twenty-one grafts (33.8%) were lost and 18 recipients (29.0%) were dead. Moreover, malignancy recurrence was the most prevalent reason for patient death (38.8%). As in our study, several other reports have similarly validated that the hazard for tumor recurrence after liver transplant is increased in patients with previous malignancy beyond the Milan criteria and UCLA criteria or with vascular invasion [36, 37]. Thus, for lower tumor recurrence rate after liver transplant, it is probably wise to stick to the Milan criteria for the selection of HCC patients.

There are several potential limitations to this study. Starting with the retrospective study, the cases of LT in two groups were few and this may influence the outcomes. Additionally, the follow-up time was short to compare long-term outcomes. Moreover, in this study, the cost of hospital expenses was not compared between the DCD and DBD groups. In general, the cost difference between the DCD and DBD groups could highlight the cost of treatment of morbidities between both the group recipients.

In conclusion, although the number of cases was small to some extent, this single-center study of China first reported that LT from DCD donors showed similar short- and long-term outcomes with DBD donors. It justified the widespread implementation of voluntary citizen-based deceased organ donation in China.

## Figures and Tables

**Table 1:** Characteristics of donors.

	DBD (*n* = 27)	DCD (*n* = 35)	Total (*n* = 62)	*P* value
Age, years	43 (28-47)	40 (30-45)	42 (28-46)	0.819
Male	24 (88.9)	26 (74.3)	50 (80.6)	0.149
BMI, kg/m^2^	22.5 (21.8-24.8)	22.5 (20.8-24.6)	22.5 (20.8-24.8)	0.599
Cause of death				0.277
Trauma	16 (59.3)	20 (57.1)	36 (58.1)	
Stroke	9 (33.3)	10 (28.6)	19 (30.6)	
Brain tumor	0 (0)	4 (11.4)	4 (6.5)	
Anoxia	2 (7.4)	1 (2.9)	3 (4.8)	
Cardiac arrest	4 (14.8)	7 (20.0)	11 (17.7)	0.742
Duration of ICU stay, days	10 (6-16)	6 (4-10)	8 (4-13)	0.010
Serum sodium, mmol/L	151 (141-160)	159 (148-168)	156 (142-164)	0.215
Serum bilirubin, *μ*mol/L	22 (16-34)	23 (13-37)	23 (15-34)	0.855
Serum creatinine, *μ*mol/L	96 (58-167)	90 (63-132)	91 (59-143)	0.576

Data are expressed as median (interquartile range) or number (%). DCD: donation after circulatory death; DBD: donation after brain death; BMI: body mass index; ICU: intensive care unit. Note: the cardiac arrest of DCD donors occurred before the withdrawal of life support.

**Table 2:** Characteristics of recipients.

	DBD (*n* = 27)	DCD (*n* = 35)	Total (*n* = 62)	*P* value
Age, years	54 (48-58)	51 (44-55)	52 (46-57)	0.188
Male	23 (85.1)	29 (82.9)	52 (83.9)	1.000
BMI, kg/m^2^	24.2 (21.6-25.7)	21.9 (21.1-23.7)	22.5 (21.2-25.2)	0.156
Primary disease				0.159
HCC	12 (44.4)	19 (54.3)	31 (50.0)	
HBV-related cirrhosis	4 (14.8)	8 (22.9)	12 (19.4)	
Acute liver failure	3 (11.1)	2 (5.7)	5 (8.0)	
Alcoholic cirrhosis	4 (14.8)	0 (0)	4 (6.5)	
Other	4 (14.8)	6 (17.1)	10 (16.1)	
HCC beyond Milan criteria	7 (25.9)	9 (25.7)	16 (25.8)	0.779
MELD score	15 (10-19)	11 (9-18)	12 (9-19)	0.083

Data are expressed as median (interquartile range) or number (%). DCD: donation after circulatory death; DBD: donation after brain death; BMI: body mass index; HCC: hepatocellular carcinoma; HBV: hepatitis B virus; MELD: model for end-stage liver disease.

**Table 3:** Operative and postoperative characteristics of recipients.

	DBD (*n* = 27)	DCD (*n* = 35)	Total (*n* = 62)	*P* value
Donor warm ischemia time, min	0	16 (11-18)	-	-
Implantation warm ischemia time, min	35 (26-42)	53 (35-68)	41 (30-66)	0.004
Cold ischemia time, min	265 (240-309)	288 (238-326)	274 (239-314)	0.787
Anhepatic phase, min	54 (47-61)	76 (56-90)	59 (50-87)	0.007
Operation time, min	412 (347-488)	430 (364-497)	422 (355-493)	0.447
Blood loss, L	2.5 (1.2-3.2)	2.0 (1.2-4.5)	2.0 (1.2-4.0)	0.960
RBC transfusion, units	12 (5-18)	12 (6-18)	12 (6-18)	0.644
Ventilation, hours	15 (12-32)	16 (12-69)	15 (12-48)	0.366
Duration of ICU stay, days	1 (1-3)	4 (3-7)	3 (1-6)	0.001
Length of postoperative hospital stay, days	17 (14-25)	25 (16-32)	19 (15-32)	0.208
Complication grade				0.379
I/II	5 (18.5)	5 (14.3)	10 (16.1)	
III	5 (18.5)	13 (37.1)	18 (29.0)	
IV	5 (18.5)	3 (8.6)	8 (12.9)	
V	3 (11.1)	5 (14.3)	8 (12.9)	
Severe complications (≥grade III)	13 (48.1)	21 (60.0)	34 (54.8)	0.352

Data are expressed as median (interquartile range) or number (%). DCD: donation after circulatory death; DBD: donation after brain death; RBC: red blood cells; ICU: intensive care unit.

**Table 4:** Postoperative complications of recipients.

	Total (*n* = 62)
Postoperative course	
Pleural effusion	24 (38.7)
Pulmonary infection	11 (17.7)
Ascites	6 (9.7)
Renal dysfunction	6 (9.7)
Abdominal hemorrhage	6 (9.7)
Acute rejection	6 (9.7)
Incision infection	3 (4.8)
HAT	3 (4.8)
Portal vein thrombosis	1 (1.6)
PNF	1 (1.6)
IPF	1 (1.6)
Bile leakage	1 (1.6)
GVHD	2 (3.2)
Intestinal perforation	2 (3.2)

Data are expressed as median (interquartile range) or number (%). HAT: hepatic artery thrombosis; PNF: primary nonfunction; IPF: initial poor function; GVHD: graft-versus-host disease.

**Table 5:** Causes of recipient's death.

	Total (*n* = 18)
Pulmonary insufficiency	2 (11.1)
Renal dysfunction	1 (5.6)
HAT	3 (16.6)
PNF	1 (5.6)
IPF	1 (5.6)
GVHD	1 (5.6)
Malignancy recurrence	7 (38.8)
New-onset tumor	1 (5.6)
Pneumocystis carinii pneumonia	1 (5.6)

Data are expressed as median (interquartile range) or number (%). HAT: hepatic artery thrombosis; PNF: primary nonfunction; IPF: initial poor function; GVHD: graft-versus-host disease.

## Data Availability

All the data supporting the results are shown in the paper and are available from the corresponding author.
